# Bond Strength of White Mineral Trioxide Aggregate with and without Disodium Hydrogen Phosphate with Different Liquid-to-Powder Ratios

**DOI:** 10.22037/iej.v12i3.15600

**Published:** 2017

**Authors:** Hadi Mokhtari, Sara Jafarizadeh, Hamid Reza Mokhtari Zonouzi, Mehrdad Lotfi, Mohammad Forough Reyhani, Aydin Sohrabi

**Affiliations:** a *Department of Endodontics, Dental School, Tabriz University of Medical Sciences, Tabriz, Iran; *; b *Department of Orthodontics, Dental School, Tabriz University of Medical Sciences, Tabriz, Iran*

**Keywords:** Disodium Hydrogen Phosphate, Mineral Trioxide Aggregate, Push-Out Test, Root Canal Filling Materials, Root Canal Therapy

## Abstract

**Introduction:**

Mineral trioxide aggregate (MTA) can be used in the treatment of irritated vital pulp and repair of root perforations. However, the initial reaction of inflammatory cells to this material and also its setting time are not ideal. Studies have shown that disodium hydrogen phosphate (DHP), decreases the setting time of MTA, with no effect on its pH. This study was undertaken to evaluate the effect of DHP on push-out bond strength of MTA at different liquid-to-powder ratios.

**Methods and Materials:**

A total of 120 samples were prepared from the middle third of the roots of single-rooted teeth for evaluation of push-out bond strength. The push-out bond strength was measured in both groups after 72 h at different liquid-to-powder ratios, including 0.33:1, 0.5:1 and 0.6:1. Factorial ANOVA and Tukey’s HSD post-hoc tests were used to compare the differences between the independent groups. Statistical significant was set at *P*<0.05.

**Results:**

The push-out bond strengths of pure MTA and MTA+DHP groups were 10.96±5.78 and 13.32±5.03, respectively. Tukey’s HSD post-hoc test revealed significant differences between the two groups. Furthermore, there were no interactive effect between material and the liquid: powder ratio.

**Conclusion:**

Incorporation of DHP into MTA resulted in an increase in push-out bond strength of MTA, and an increase in liquid-to-powder ratio resulted in a decrease in push-out bond strength.

## Introduction

Mineral trioxide aggregate (MTA) was introduced for sealing the communication between the root canal system and the external surface of the tooth [1]. It is widely used due to its favorable properties, including dimensional stability, biocompatibility, good sealing ability, relative antibacterial effect on *Enterococcus faecalis* and ability to set in the presence of blood and moisture [2-11]. MTA is used in the treatment of vital pulp and repair of root perforations. It can also be used as an apical plug in immature necrotic teeth and as a root-end obturation material in apical surgeries [12]. In addition, endodontic microsurgery with MTA has exhibited a higher success rate compared to more conventional materials and techniques [13]. However, the initial reaction of inflammatory cells to this material [14] and also its setting time [15] are not favorable. 

Various materials and techniques have been used to improve the properties of MTA. One of these materials is disodium hydrogen phosphate (DHP) [3]. Lotfi *et al. *[16] reported that incorporation of DHP into white MTA resulted in better biocompatibility, compared to pure white MTA, in the subcutaneous tissues of rats.

In another study, Haung *et al. *[3] showed that incorporation of DHP into MTA resulted in a significant decrease in its setting time to 26 min. In addition, incorporation of DHP into MTA resulted in an increase in its tensile strength during the first few hours from 1 MPa to 4.9 MPa, despite maintaining its initial pH (11) and final pH (13.2). They suggested that DHP can be used as an accelerator for the setting reaction of MTA cement.

The bond strength is an important factor in providing a favorable seal between the root canal system and the external surface of the root [17]. One of the techniques used to evaluate resistance of dental materials to dislodgment is the push-out test [18]. An ideal endodontic material should resist mechanical forces during tooth function or tooth restoration and should not be dislodged [19, 20]. However, MTA might be dislodged in some clinical situations such as the repair of furcal perforations or direct pulp capping procedures due to coronal forces that are exerted during restoration of the tooth [21].

It has been shown the push-out bond strength of MTA to dentin are affected by moisture [22], acidic [23], or alkaline environment [24], different root canal irrigants [25], calcium hydroxide [5] and some additives to MTA [21].

When Portland cement is mixed with water, a special structure is formed, which is composed of micropores, capillary canals and trapped water; when the liquid-to-powder ratio increases, there is an increase in the porosity of the mixture [26]. Therefore, the water content of the mixture is an important determinant of the material’s properties because the porosity of the MTA mixture is one of the factors affecting its push-out bond strength.

No studies to date have evaluated the effect of incorporating DHP into MTA at different liquid-to-powder ratios on its push-out bond strength.

## Materials and Methods

A total of 120 dentin discs were prepared from the middle third of the roots of teeth with only one root canal for the evaluation of push-out bond strength. First the crown was removed in all the teeth. Then the middle third of each remaining root was cut using a diamond disk at low speed under a water coolant in order to prepare samples with smooth cross-sections, measuring 2 mm in thickness. At this stage cross-sections with ovoid root canals were excluded from the study. Then the root canal space in each sample was enlarged using a #5 Gates-Glidden drills (Foshan Duoyimei Medical Instrument Co, Ltd, China) using the Van der Weele technique [23] in order to create standard cavities measuring 1.3 mm. Then the diameter of each cavity was measured and those with root canal diameters over 1.3 mm and those with inadequate thickness of dentin around the root canal were excluded from the study. A pilot study was carried out to determine proper liquid-to-powder ratios and based on the results of this study and the recommendations of the manufacturer, the liquid-to-powder ratios of 0.33:1, 0.5:1 and 0.6:1 were determined and used in 6 groups (*n*=20) as follows: Group A1: a mixture of 1 g white MTA powder (Angelus, Londrina, PR, Brazil) and 0.33 mL of liquid; Group A2: a mixture of 1 g white MTA powder and 0.5 mL of liquid; Group A3: a mixture of 1 g white MTA powder and 0.6 mL of liquid; Group B1: a mixture of 1 g white MTA powder plus 0.18 g of DHP and 0.33 mL of liquid; Group B2: a mixture of 1 g white MTA powder plus 0.18 g of DHP and 0.5 mL of liquid and Group B3: a mixture of 1 g white MTA powder plus 0.18 g of DHP and 0.6 mL of liquid.

Before mixing white MTA with DHP and its liquid, all the study materials, molds, spatulas and glass slabs were placed at room temperature for 24 h. 

The samples were first immersed in 17% ethylene diamine tetraacetic acid (EDTA) solution for 3 min, followed by immersion in 1% NaOCl solution for another 3 min. Then the samples were rinsed in distilled water and dried to some extent. A total of 7.5 g of white MTA powder and 0.18 g of DHP were placed in an empty amalgam capsule and mixed in an amalgamator for 60 sec to achieve a homogeneous mixture. Then the cement was prepared by accurate measurement of the powder with the use of a weighing machine and the liquid with the use of a micropipette; mixing was carried out using the manual technique. 

In each subgroup, the cement was carried into the root canal lumen using a carrier and condensed within the cavities with a plugger. The root segments were wrapped in gauze pieces impregnated with synthetic tissue fluid and incubated at 37^°^C for 72 h. The synthetic tissue fluid was prepared using 0.17 g of KH_2_PO_4_, 1018 g of Na_2_HPO_4_, 8 g of NaCl and 0.2 g of KCl, mixed with 1 g of water (pH=7.4) using the technique introduced by Saghiri [24]. Then the push-out bond strength values, which represented the bond strength of the samples to dentin, were measured in MPa using a universal test machine (Hounsfield Test Equipment, model HSK-S, Surrey, UK). The samples were first placed on a metallic slab with a central cavity in this machine so that its plunger would move freely. Then a compressive force was applied on MTA surface with the use of a stainless steel plunger, measuring 1 mm in diameter, at a crosshead speed of 1 mm/min.

The diameter of the plunger was 0.3 mm smaller than the diameter of the prepared cavities; therefore, the approximate distance between the plunger and the tooth surface was 0.1-0.15 mm to ensure a complete contact with the samples. To calculate the push-out bond strength in MPa in each sample, the maximum force applied to MTA surface at dislodgment was divided by the contact surface area. The 2*πr*×*h *formula was used to calculate the contact surface area where *π* is the constant (3.14), *r* is the radius of the root canal and *h* is the thickness of the root sample in mm. 

Factorial ANOVA and Tukey’s HSD post-hoc tests were used to compare the differences in the means of independent groups. Statistical significance level was defined at 0.05. 

## Results

Table 1 presents the descriptive statistics of push-out bond strength tests. The results of ANOVA test showed that the type of the material used in preparation of the cavity had a significant effect on its push-out bond strength (*P*=0.005). Factorial ANOVA was used to evaluate the effects of different liquid-to-powder ratios of MTA cement on the push-out bond strength. The results showed statistically significant differences between the groups (*P*<0.001).

Post hoc Tukey’s HSD test was used for two-by-two comparison of the subgroups. Based on the results, there were no significant differences between 0.5:1 and 0.6:1 liquid-to-powder ratio (*P*=0.078); however, there were significant differences between the 0.33:1 liquid-to-powder ratio and the two other ratios (*P*<0.001 and *P*<0.001). Furthermore, based on the results of factorial ANOVA, incorporation of DHP into MTA cement and use of lower liquid-to-powder ratios had no interactive effect on the push-out bond strength of MTA cement (*P*=0.94).

## Discussion

When modifications are made in the basic formulation of an endodontic material, all the physical, antimicrobial, sealing and biocompatibility properties of the new material should be evaluated before its clinical application. Therefore, before the use of DHP as an accelerator for the setting reaction of MTA, its effect on other properties of this cement, including its bond to dentin, should be evaluated. 

To evaluate the push-out bond strength in the present study, a standard, effective and reliable technique was used. A recent study used finite element analysis to evaluate the limitations of the push-out bond strength test and concluded that this test results in parallel fracture of the dentin-cement interface and is an effective test for the evaluation of bond strength. The results of that study suggested that the ratio of the test tool plugger diameter to the diameter of MTA filling should be under 0.85 and the thickness of root sections should be 1.1 mm; in the present study the ratio mentioned above was 0.84 and the thickness of all the sections was 2 mm [27, 28]. 

The results of the present study showed that the liquid-to-powder ratio of Angelus MTA cement affects its push-out bond strength and incorporation of DHP results in an increase in its push-out bond strength. Kogan *et al.* [15] showed that adding DHP to MTA act only as an accelerator and it did not effect on the compressive strength; but in the present study, adding DHP leads to an increase in the push-out strength. DHP can increase the amount and properties of hydration products [29]. The push-out bond strength values achieved in the present study confirmed the results of studies by Reyes-Carmona* et al. *[30] and Lotfi *et al. *[17]. Reyes-Carmona *et al. *[30] revealed that the biomineralization process begins after contact of dentin with MTA, resulting in the controlled distribution of apatite between cement and dentin, which is responsible for the primary chemical bond between them [30]. In the present study, too, incorporation of DHP into the cement itself improves the biomineralization process in a similar manner. Previous studies have shown that MTA cement has physical properties similar to those of the Portland cement [31]. 

**Table1:** Descriptive statistics of push-out bond strengths of the samples in MPa

**Group**	**Mean (SD)**		
	**0.33:1**	**0.5:1**	**0.6:1**
**MTA**	15.72 (5.92)	9.98 (4.85)	7.6 (3.25)
**MTA+DHP**	17.8 (4.76)	11.96 (3.87)	10.21 (2.79)

*Different letter in each column indicates statistically significant differences*

In the present study, the early push-out strength of MTA and MTA mixed with Na_2_HPO_4 _was evaluated after 72 h. Acceleration of hydration process affects the physical properties of the set MTA cement [32]. The hydration process and nucleation of hydrated calcium silicate are responsible for the mechanical strength of MTA cement [33]. Therefore, it can be concluded that incorporation of DHP increases the push-out bond strength by increasing hydration and production of hydroxyapatite. The reaction of MTA cement with water results in the production of silicate cement and calcium hydroxide, and the reaction of calcium hydroxide with DHP results in the formation of tricalcium phosphate or hydroxyphosphate, the deposition of which in the dentinal tubules and at the dentin-restorative material interface results in an increase in push-out bond strength.

## Conclusion

It can be concluded under the limitations of the present study that incorporation of DHP into MTA results in an increase in its push-out bond strength, and an increase in the liquid-to-powder ratio decreases its push-out bond strength.
